# Effect of postoperative Trendelenburg position on shoulder pain after gynecological laparoscopic procedures: a randomized clinical trial

**DOI:** 10.1186/s12871-020-0946-9

**Published:** 2020-01-29

**Authors:** Carine Zeeni, Dina Chamsy, Ali Khalil, Antoine Abu Musa, Majed Al Hassanieh, Fadia Shebbo, Joseph Nassif

**Affiliations:** 1 Department of Anesthesiology, American University of Beirut Medical Center, P.O. Box 11-0236, Beirut, Lebanon; 2 Department of Obstetrics and Gynecology, American University of Beirut Medical Center, Beirut, Lebanon

**Keywords:** Trendelenburg position, Shoulder pain, Laparoscopic surgery, Gynecological surgery

## Abstract

**Background:**

Laparoscopic surgery has become a standard of care for many gynecological surgeries due to its lower morbidity, pain and cost compared to open techniques. Unfortunately, the use of carbon dioxide (CO_2_) to insufflate the abdomen is the main contributor to post-operative shoulder pain.

**Methods:**

We aim to assess the effect of postoperative Trendelenburg position on shoulder pain after gynecological laparoscopic procedures. We hypothesize that maintaining the patient in Trendelenburg for 24 h postoperatively will significantly decrease postoperative shoulder pain and analgesic consumption. After obtaining written informed consent, 108 patients were prospectively randomized into two groups. In the control group, patients underwent standard gynecologic laparoscopic procedures; then after passive deflation of the pneumoperitoneum at the end of the surgery, the patients were placed in supine head up position in the post anesthesia care unit (PACU) and received our institution’s common postoperative care. Patients in the intervention group were subjected to the same maneuver but were positioned in a Trendelenburg position (20 °) once fully awake and cooperative in the PACU and retained this position for the first 24 h. Numerical rating scale (NRS) was used to assess shoulder pain and nausea upon patient arrival to the PACU, at 4, 6, 12 (primary outcome) and 24 h postoperatively. Time to first rescue pain medication, total rescue pain medications and overall satisfaction with pain control were recorded. 101 patients were included in the final data analysis.

**Results:**

Both groups were comparable in terms of baseline characteristics. NRS pain scores were significantly lower in the intervention group at 12 h compared to the control group (0 [0–1] versus 5 [1–4], *p* < 0.001), furthermore improvement in postoperative shoulder pain between time of arrival to PACU (time zero) and 12 h postoperatively was significantly higher in patients allocated to the experimental group compared to the control group. Pain scores were significantly lower in patients allocated to the experimental group versus the control group (0 [0–1] versus 5 [1–4], *p* < 0.001).

**Conclusion:**

In conclusion, Trendelenburg position is an easy non-pharmacologic intervention that is beneficial in reducing postoperative shoulder pain following gynecologic laparoscopic surgery.

**Trial registration:**

Retrospectively registered at Clinicaltrials.gov, registration number NCT04129385,  date of registration: June 28, 2019

## Background

Laparoscopic gynecologic surgery has evolved from a limited surgical procedure used only for diagnostic purposes to a major surgical approach for treating a multitude of malignant and non-malignant pathologies. It is currently one of the most common surgical procedures performed by gynecologists [1]. Although laparoscopic surgery has proven its superiority over laparotomy in terms of improved post-operative pain scores, postoperative shoulder pain remains a major concern following laparoscopic surgeries. Shoulder pain is reported to occur in 35 to 70% of laparoscopic surgeries [2, 3]. The pain can be severe and is usually relieved in 24–48 h, but rarely persists for over 72 h after surgery [4]. The precise mechanism of this shoulder pain remains unclear. The main hypothesis is the presence of residual carbon dioxide (CO_2_) in the abdominal cavity that causes irritation of the phrenic nerve and referred pain to the shoulders [5, 6]. Other theories include peritoneal stretching, diaphragmatic irritation or injury, and shoulder abduction during surgery [7–9].

Various preventative measures have been proposed intraoperatively to try to reduce residual CO_2_ in the abdominal cavity including: low insufflation rate and pressure [10], Valsalva maneuvers [11, 12], filling the abdominal cavity with Lactated Ringers [13], and active deflation of the abdomen [14]. To our knowledge, there are no available published studies looking at the effect of postoperative Trendelenburg positioning on the incidence of shoulder pain after laparoscopic gynecologic surgery. The Trendelenburg position might decrease pain by reducing the mechanical pressure exerted by CO_2_ on the diaphragm and the upper abdominal muscles. CO_2_, known for its high solubility, would also be displaced to the pelvis that has a rich vasculature which in turn speeds up the resorption of pneumoperitoneum.

The purpose of this study is to assess the effect of postoperative Trendelenburg position on shoulder pain after gynecologic laparoscopic procedures. We hypothesize that maintaining the patient in Trendelenburg for 24 h postoperatively will significantly decrease postoperative shoulder pain.

## Materials and methods

### Subjects and study design

**Fig. 1:**
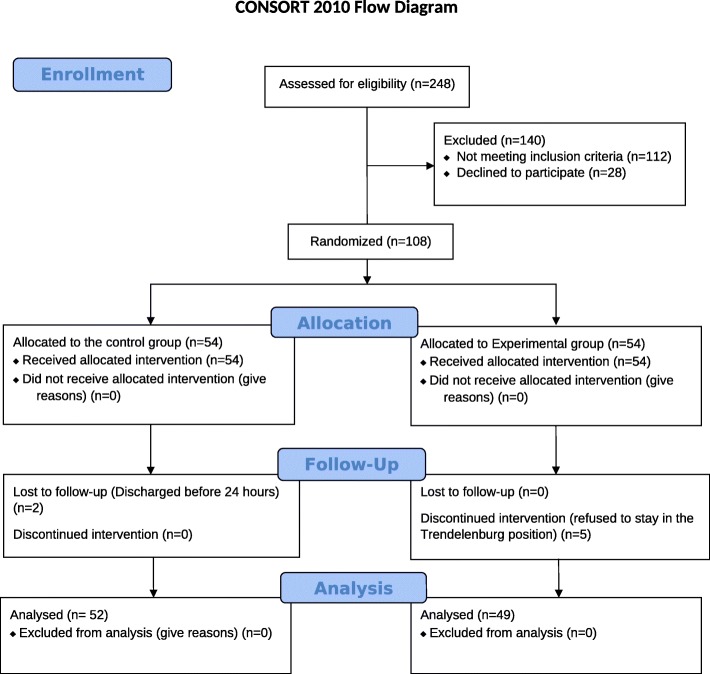
Consort flow diagram

This is a prospective randomized controlled study that was conducted at the American University of Beirut Medical Center (AUBMC), on patients undergoing laparoscopic gynecologic surgeries. This study was approved by AUBMC’s Institutional Review Board (IRB ID: OGY.JN.03) and written informed consent was obtained from all patients. The study adheres to the CONSORT guidelines (Fig. 1) and was retrospectively registered at clinicaltrials.gov  (NCT04129385, principal investigator: Joseph Nassif, date of registration: June 28, 2019).

This study included female patients, aged between 18 and 60 years, with American Society of Anesthesiologist (ASA) physical status I or II scheduled for diagnostic or operative gynecological laparoscopic surgery of one to three-hour duration with abdominal incisions measuring less than 1.6 cm in size. Patients with the following criteria were excluded: conversion of the surgery to laparotomy, requirement of an abdominal insufflation pressure greater than 14 mmHg, history of gastro-esophageal reflux, thrombophilia or high risk of deep vein thrombosis according to the ACOG 2007 practice bulletin, pregnancy, morbid obesity (BMI > 40), and 1 day surgery. Patients were randomly allocated to Groups 1 (Control) and 2 (Intervention) using a computer-generated randomization table. Blinding of the group allocation was not possible due to the design of the study.

### Study design

All patients received Thrombo-Embolic-Deterrent (TED) stockings preoperatively. Intravenous (IV) access was established in the induction room then standard ASA monitoring devices were applied in the operating room. Induction of anesthesia was achieved using midazolam 1–2 mg fentanyl 1–2 μg/kg, lidocaine 1.5 mg/kg, and propofol 2 mg/kg IV. Rocuronium 0.6 mg/kg was administered to facilitate tracheal intubation. All patients received dexamethasone 8 mg IV after induction to prevent postoperative nausea and vomiting (PONV). Maintenance of anesthesia was provided using a mixture of oxygen and air (FiO_2_ = 50%), sevoflurane (1–1.2 MAC), fentanyl and rocuronium.

Upon deflation of abdomen, fentanyl 1 μg/kg was given for postoperative pain relief and ondansetron 4 mg for PONV prevention. At the end of surgery, muscle relaxation was reversed with a combination of glycopyrrolate/neostigmine or sugammadex.

In the control group, patients underwent the standard laparoscopic procedure. While still in Trendelenburg position and prior to wound closure and with the laparoscopic port valves open, the patients’ abdomen was passively deflated. The patients were placed in supine head up position in the post anesthesia care unit (PACU) and postoperatively as is common practice at our institution. Patients in the intervention group were subjected to the same maneuver as the control group patients prior to wound closure but were positioned in a Trendelenburg position (20 °) once fully awake and cooperative in the PACU. They retained this posture for the first 24 h postoperatively. The maximum time allowed in a straight-up position was three 15-min intervals over a 24-h period (the first interval being at the time of clear fluid intake at 12 h postoperatively).

Incentive spirometry was mandatory for all patients postoperatively once fully awake.

Postoperative pain and nausea management was standardized and provided systematically for all patients. Starting in the PACU, medications included intravenous administration of 1 g acetaminophen IV and 100 mg ketoprofen IV every 6 and 8 h, respectively. Tramadol 100 mg IV was used as a rescue medication that was also given intravenously every 8 h upon demand. 4 mg of ondansetron and/or 10 mg of metoclopramide were given every 8 h as rescue medication for nausea and/or vomiting. Data collection of postoperative pain and nausea started at the arrival of patients to the PACU, then at 4, 6, 12 and 24 h postoperatively. Total amount of rescue pain and nausea medications used was recorded at all time-points.

### Outcome measures

The primary outcome of this study was the presence and severity of shoulder pain 12 h after laparoscopic surgery. The numerical rating scale (NRS) was used to assess for pain measures on a 0 to 10-point scale; 0 representing “no pain” and 10 representing “worst pain”. Secondary outcomes included the presence and severity of shoulder pain and nausea upon patient arrival to the PACU, then 4, 6, 12 and 24 h postoperatively using the NRS scale. Time to first rescue pain medication, total rescue pain medications during the first 24 h post-surgery, and the patients’ pain scores (using the NRS) with the overall satisfaction from pain control were also recorded.

### Statistical and power analysis

This is a two-sided randomized controlled study, with a proposed power of 80% and alpha = 0.05.

The sample size calculation was done by expecting a 30% reduction in shoulder pain in the interventional group compared to the control one at 12 h post operatively. Thus, a total sample size of 108 patients was obtained, divided into 54 patients in each group, taking into account a maximum dropout rate of 20%. The later rate is expected because of non-tolerance of the Trendelenburg position or to conversion to laparotomy if needed.

The Statistical Package for the Social Sciences Software (SPSS) and the Statistical Analysis System (SAS) were used for the data analysis. Data are presented as mean ± SD or median [IQR] for continuous data and frequency (percentage) for categorical data. Proc mixed test was used to the mixed group and time effect on pain and nausea scores post-operatively. Student’s t-test was used to compare the normally distributed continuous data and Mann-Whitney test was used for ordinal data. Chi-square test or Fisher exact test was used for categorical data.

## Results

A total of 248 patients were assessed for eligibility and 108 enrolled in the study between June 2016 and June 2018. Seven patients were excluded (five withdrew because they refused to stay in Trendelenburg position for the total duration of the study, and two were discharged before 24 h postoperatively). 101 patients were included in the final data analysis (52 patients in the control group and 49 patients in the experimental group).

Basic demographics, types of the surgical procedures, and procedural duration are presented in Table 1. Both were comparable with no significant differences between the two groups. We did not observe any hemodynamic or respiratory side effect that required any intervention in any of the patients throughout the study period and no patients were re-admitted due to any hemodynamic instability or respiratory adverse event.

**Table 1:** Demographic Characteristics, Types of Surgical Procedures, and Procedural Durations

	Group 1, Control (*n* = 52)	Group 2, Trendelenburg (*n* = 49)	*p*-value
Age, years	38.42 ± 10.69	36.29 ± 8.67	0.27
Weight, kg	66.96 ± 12.66	61.98 ± 11.58	0.06
Surgery type			
Laparoscopic Hysterectomy	15 (28.8)	7 (14.3)	
Laparoscopic Adnexectomy	12 (23.1)	15 (30.6)	
Laparoscopic Endometriosis	3 (5.8)	6 (12.2)	
Laparoscopic Myomectomy	12 (23.1)	14 (28.6)	
Others	10 (19.2)	7 (14.3)	
Intraoperative time, minutes	105.5 (48.2)	92.37 (36.2)	0.23
Laparoscopic time, minutes	88 (47.4)	85.88 (54.5)	0.83
Total intra-op fentanyl, mcg	281 ± 88.9	283 ± 84.31	0.91

Values are mean ± SD, number (%)

**Fig. 2:**
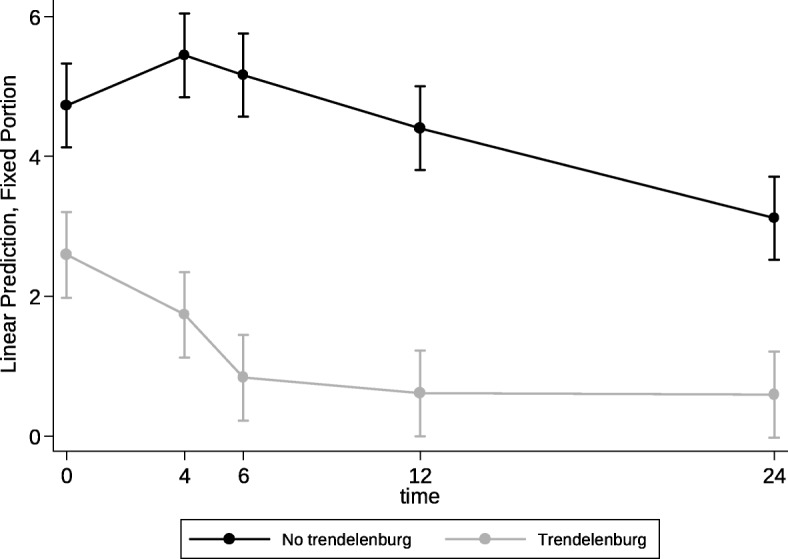
Postoperative Shoulder Pain (Numerical Rating Scale) Over Time

Pain scores were significantly lower in the Trendelenburg group, and the trend was a decreasing pain score in both groups over time (Table 2). This effect was highly significant when taking into consideration the group allocation and different time points. Improvement in postoperative shoulder pain between time of arrival to PACU (time zero) and 12 h postoperatively was significantly higher in patients allocated to the experimental group compared to the control group with pain severity decreasing by 76% compared to 6.9% (*p* < 0.001) respectively (Fig. 2).

**Table 2:** Postoperative Shoulder Pain Scores

	Group 1, Control (*n* = 52)	Group 2, Trendelenburg (*n* = 49)	*p*-value
NRS Pain Score			
PACU	6.00 [1.00–7.75]	2.00 [1.00–4.00]	0.003
4 h postop	6.00 [2.00–8.00]	2.00 [1.00–3.00]	< 0.001
6 h postop	6.00 [4.00–7.00]	1.00 [0.00–1.00]	< 0.001
12 h postop	5.00 [3.00–6.00]	0.00 [0.00–1.00]	< 0.001
24 h postop	3.00 [1.25–5.00]	0.00 [0.00–1.00]	< 0.001
NRS PONV Score			
PACU	2 [0.00–6.00]	1 [0.00–2.00]	0.033
4 h postop	2 [0.00–4.75]	1 [0.00–1.00]	< 0.001
6 h postop	1 [0.00–3.00]	0 [0.00–1.00]	0.001
12 h postop	1 [0.00–2.00]	0 [0.00–1.00]	< 0.001
24 h postop	1 [0.00–2.00]	0 [0.00–1.00]	< 0.001

*PACU* Post Anesthesia Care Unit, *PONV* Post-Operative Nausea and Vomiting

Values are medians and interquartile ranges

Time to first analgesic request was longer in the experimental group compared to the control group (111.39 ± 132.58 min vs. 85.86 ± 134.64 min, *p* = 0.46 respectively), yet the difference was not statistically significant.

Nausea scores significantly decreased with time in both groups, and was significantly higher in the experimental group (Table 2). Incidence of nausea at any time postop was not statistically different between the two groups (78% vs. 75% respectively with *p* = 0.8). However, the total PONV medications used were significantly lower in the experimental group, metoclopramide consumption (10.00 ± 14.95 mg vs. 4.08 ± 0.16, *p* = 0.016) and ondansetron consumption (0.85 ± 2.00 mg vs. 0.16 ± 0.80 mg, *p* = 0.036).

Non-opioid and opioid consumption showed statistically significant difference between both groups (Table 3). Patients allocated to the experimental group had lower postoperative analgesic consumption compared to the control group (*p* < 0.001).

**Table 3:** Opioid and Non-Opioid Postoperative Analgesic Consumption

	Group 1, Control	Group 2, Trendelenburg	*p*-value
Non-Opioid Consumption			
Acetaminophen, g	2 ± 1.4	0.99 ± 1.41	< 0.001
Ketoprofen, mg	146.15 ± 105.64	44.89 ± 86.75	< 0.001
Opioid consumption, mg	8.12 ± 7.07	0.82 ± 2.76	< 0.001
Time to first analgesic request, min	85.86 ± 134.64	111.39 ± 132.58	0.46

Values are mean ± SD

*total amount of opioids consumed (Tramadol and Morphine, where Tramadol was converted to morphine equivalent doses)

**Fig. 3:**
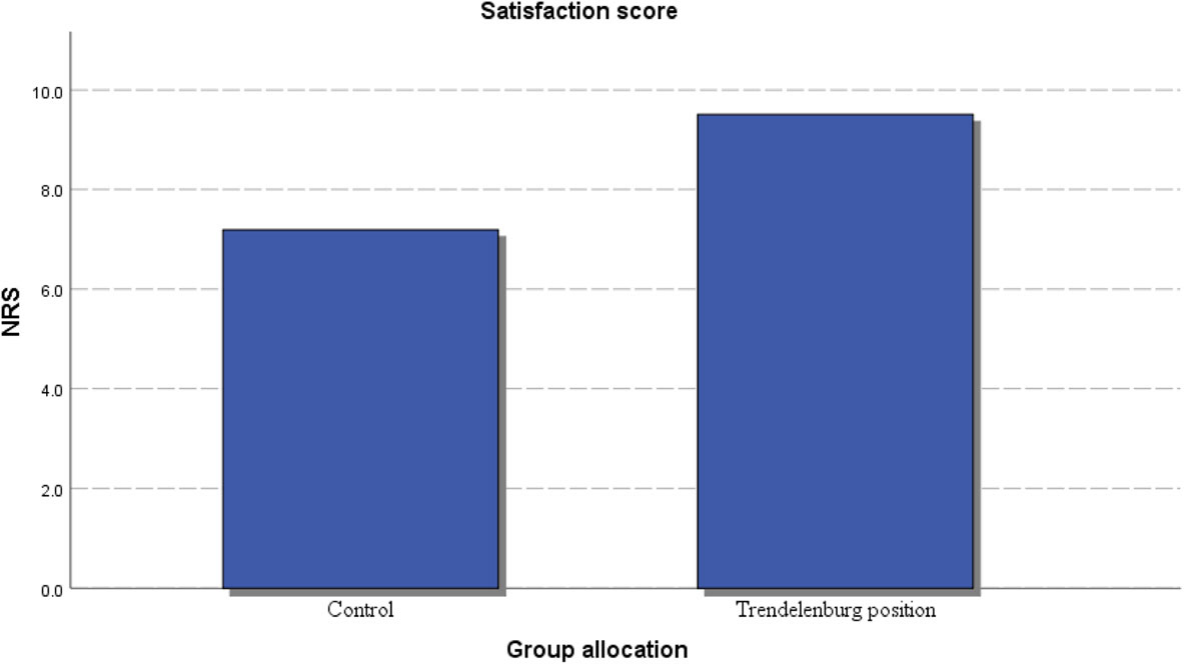
Patient Overall Satisfaction Score from pain control

Satisfaction score was significantly higher in patients who were randomized to the Trendelenburg position (*p* < 0.001). These patients had a median score of 9.5 compared to a score of 8 in the control patients (Fig. 3).

## Discussion

As previously noted, shoulder pain is reported to occur in 35 to 70% of laparoscopic surgeries [2, 3], mostly to the patients’ right side. The phrenic nerve originates from the C3 to C5 cervical nerves in the neck and descends through the thorax to innervate the diaphragm. A link between phrenic nerve irritation and this referred type of pain is suggested in the literature [15, 16]. Severe postoperative shoulder pain may lead to patient dissatisfaction but also to pulmonary complications such as atelectasis and pneumonia as patients are unable to take deep breaths. This study supports the theory that Trendelenburg position displaces retained CO_2_ gas towards the pelvis and away from the diaphragm, thereby decreasing phrenic nerve irritation and hence shoulder pain, as well as a quicker resorption of the soluble CO_2_ gas in a highly vascular area which is the pelvis.

This study is the first to assess the effect of Trendelenburg position on postoperative shoulder pain following laparoscopic gynecologic surgery. Only one other study by Aydemir et al. [15] looked prospectively at the effect of Trendelenburg position on postoperative shoulder pain, however the study subjects were patients undergoing laparoscopic cholecystectomy.

It is difficult to compare both studies because they are not identically designed. This study is prospective and randomized while the other is non-randomized. Moreover, the duration of the study intervention (Trendelenburg positioning) as well as the study subjects and the nature and duration of the surgical procedures are different. While this study required that patients be placed in Trendelenburg position for 24 h postoperatively and measured the pain scores at 4, 6, 12 and 24 h, the study by Aydemir et al. placed patients in extreme Trendelenburg position upon reporting shoulder pain for only 10 min at a time, and recorded pain scores 10 min afterwards. The degree of Trendelenburg was not mentioned. Pain scores were statistically significantly improved thereby supporting the theory that Trendelenburg position decreases the phrenic nerve irritation caused by the CO_2_ gas. Similar to this study results, total analgesic consumption over 24 h was statistically significantly improved in the experimental compared to the control group.

Aydemir et al. demonstrated that Trendelenburg position is both quick and effective, with improvement in pain scores as early as ten minutes after Trendelenburg positioning. Acute and fast improvement of shoulder pain was supported by our study as the most acute drop in shoulder pain score was noted from 0 to 6 h (Fig. 2). Beyond 6 h, the pain score was maintained more or less at the same level and did not improve further. Since some patients are unable to tolerate Trendelenburg position for a long time, adopting it for a shorter period of time may be enough to significantly improve shoulder pain scores. Further studies are needed to determine the optimal duration of this intervention for shoulder pain management.

Many other methods to decrease postoperative shoulder pain have been described in the literature. The most recent Cochrane review by Kaloo et al. [16] reviewed all interventions mentioned in the literature on shoulder pain following laparoscopic gynecologic surgery. Trendelenburg position is not listed as one the possible interventions in this review article. Among all the described methods, authors concluded that potentially beneficial interventions in the reduction of postoperative shoulder pain include: a specific technique for releasing the pneumoperitoneum (such as pulmonary recruitment maneuvers, extended assisted ventilation or active aspiration of intra-abdominal gas), intraperitoneal fluid instillation, placement of an intraperitoneal drain and local anesthetic application into the peritoneal cavity (not sub diaphragmatic). Comparing these interventions to postoperative Trendelenburg positioning through randomized controlled trials is important to evaluate which of them all is most beneficial, and which carries the lowest risk of adverse events.

This randomized study provides solid evidence that the intervention is beneficial in reducing shoulder pain. One limitation is that although all healthcare providers and patients were blinded to the study intraoperatively, they were not blinded to the patient postoperatively hence patients might have underreported pain when in the Trendelenburg position and there might have been a small bias due to a placebo effect. Another limitation is the duration of the study intervention: Although most patients tolerated Trendelenburg positioning for 24 h, six refused to stay in Trendelenburg for the entire 24 h and hence withdrew from the study, but the rate of dropout between the two groups was not significant and was below the 20% expected level. Moreover, among the advantages of laparoscopic surgery are early or immediate resumption of regular diet, early ambulation and short hospital stay including same day discharge when applicable. Patients in the intervention arm were kept on a clear fluid diet for 12 h postoperatively, could not ambulate immediately postoperatively and were not discharged till after 24 h, thereby limiting some of the advantages of minimally invasive surgery. However, we can suggest to keep this position as much as feasible at home if an earlier discharge can be suggested in the future.

## Conclusions

In conclusion, Trendelenburg position is an easy non-pharmacologic intervention that is beneficial in reducing postoperative shoulder pain following gynecologic laparoscopic surgery, decreasing the amount of analgesic consumption and improving patients’ overall satisfaction with the surgical experience. Being non-pharmacologic, it can be administered by trained nursing staff and can even be taught to patients and implemented at home by simply elevating the pelvis with the use of pillows. Not only does it have zero cost, it can potentially decrease medical expenses as less analgesics are administered. More importantly, the smaller the amount of analgesic consumption, the lower the risk of medication adverse events such as respiratory depression, nausea, pruritus and ileus which are often encountered with the use of opioids [15]. Additional studies are required to determine whether Trendelenburg positioning improves postoperative shoulder pain following non-gynecologic procedures and to delineate the optimal duration of this intervention to maximally decrease shoulder pain scores.

## Data Availability

The datasets used and/or analyzed during the current study are available from the corresponding author on reasonable request.

## Abbreviations

ASA: American Society of Anesthesiologists
NRS: Numerical Rating Scale
PACU: Post Anesthesia Care Unit
PONV: Postoperative Nausea and Vomiting
TED: Thrombo-Embolic-Deterrent
